# Supplementary material for: Complexome profiling of the Chlamydomonas psb28 mutant reveals TEF5 as an early PSII assembly factor
Source: Plant Cell. 2025 Mar 17;37(6):koaf055. doi: 10.1093/plcell/koaf055 (PMC12164590; doi:10.1093/plcell/koaf055)
Supplement: koaf055_Supplementary_Data [file koaf055_supplementary_data.zip › psb28_interactive.zip!/plots/intensity/qualityPlots/peptides/FTT2_Cre06.g257500.t1.2.html]

### Cre06.g257500.t1.2

Trivial name: FTT2  
SeqCoverage [median]: 44.50  
Mass: 28.00 kDa  
  
 

| Sequence | MissedCleavage | Mass [Da] | wtIntensity | muIntensity | Score |
| --- | --- | --- | --- | --- | --- |
| AAAAELTLAAYK | 0 | 1191.65 | 4222.43 | 31761.18 | 123.63 |
| ADELPTTNPIR | 0 | 1225.63 | 0.00 | 3121.78 | 68.73 |
| AEANSRELALYSAK | 1 | 1521.78 | 0.00 | 1901.30 | 48.00 |
| ELALYSAK | 0 | 893.49 | 5794.33 | 8533.80 | 110.81 |
| IAEQSER | 0 | 831.41 | 2570.31 | 3669.96 | 150.84 |
| ILQSIEQSEQAK | 0 | 1372.72 | 3647.13 | 5085.69 | 192.50 |
| ILQSIEQSEQAKGNEKR | 2 | 1957.02 | 2613.43 | 5257.45 | 218.12 |
| NLLSVSYK | 0 | 922.51 | 1587.20 | 0.00 | 63.56 |
| VAQEKADELPTTNPIR | 1 | 1780.93 | 803.45 | 799.29 | 46.89 |
| VAVLANEQELSVEER | 0 | 1684.86 | 3742.15 | 11698.64 | 225.55 |
| YLSEYKTDEAK | 1 | 1345.64 | 821.02 | 3600.80 | 94.79 |
| YQDMVEEMKK | 1 | 1299.58 | 545.81 | 2366.19 | 83.18 |
|  |  |  | 26347.3 | 77796.1 |  |
